# proGenomes4: providing 2 million accurately and consistently annotated high-quality prokaryotic genomes

**DOI:** 10.1093/nar/gkaf1208

**Published:** 2025-11-20

**Authors:** Anthony Fullam, Ivica Letunic, Oleksandr M Maistrenko, Alexandre Areias Castro, Luis Pedro Coelho, Anastasiia Grekova, Christian Schudoma, Supriya Khedkar, Mahdi Robbani, Michael Kuhn, Thomas S B Schmidt, Peer Bork, Daniel R Mende

**Affiliations:** Structural and Computational Biology Unit, European Molecular Biology Laboratory, Heidelberg 69117,Germany; Biobyte solutions GmbH, Bothestraße 142, Heidelberg 69117,Germany; Swammerdam Institute for Life Sciences, University of Amsterdam, Amsterdam 1000 BE, The Netherlands; Centre for Microbiome Research, School of Biomedical Sciences, Queensland University of Technology, Translational Research Institute, 37 Kent St., Brisbane, Queensland, Woollongabba 4102, Australia; Centre for Microbiome Research, School of Biomedical Sciences, Queensland University of Technology, Translational Research Institute, 37 Kent St., Brisbane, Queensland, Woollongabba 4102, Australia; Structural and Computational Biology Unit, European Molecular Biology Laboratory, Heidelberg 69117,Germany; Structural and Computational Biology Unit, European Molecular Biology Laboratory, Heidelberg 69117,Germany; BioQuant, University of Heidelberg, 69120 Heidelberg, Germany; Structural and Computational Biology Unit, European Molecular Biology Laboratory, Heidelberg 69117,Germany; Structural and Computational Biology Unit, European Molecular Biology Laboratory, Heidelberg 69117,Germany; APC Microbiome and School of Medicine, University College Cork, T12 YT20 Cork, Ireland; Structural and Computational Biology Unit, European Molecular Biology Laboratory, Heidelberg 69117,Germany; Max Delbrück Centre for Molecular Medicine, Berlin 13125, Germany; Department of Bioinformatics, Biocenter, University of Würzburg, Würzburg 97074, Germany; Molecular Medicine Partnership Unit, University of Heidelberg and European Molecular Biology Laboratory, Heidelberg 69120, Germany; Human Biology-Microbiome-Quantum Research Center (WPI-Bio2Q), Keio University, Tokyo 108-8345, Japan

## Abstract

The pervasive availability of publicly available microbial genomes has opened many new avenues for microbiology research, yet it also demands robust quality control and consistent annotation pipelines to ensure meaningful biological insights. proGenomes4 (prokaryotic Genomes v4) addresses this challenge by providing a resource of nearly 2 million high-quality microbial genomes, a doubling in scale from previous versions, encompassing over 7 billion genes. Each genome underwent rigorous quality assessment and comprehensive functional annotation by applying multiple standardized annotation workflows, including the systematic identification of mobile genetic elements and biosynthetic gene clusters. proGenomes4 contains 32 887 species with ecological habitat metadata as well as precomputed pan-genomes. This substantially expanded resource provides the microbiology community with a foundation for large-scale comparative studies and is freely accessible via a newly developed command line interface and at https://progenomes.embl.de/.

## Graphical Abstract

**Graphical Abstract:**
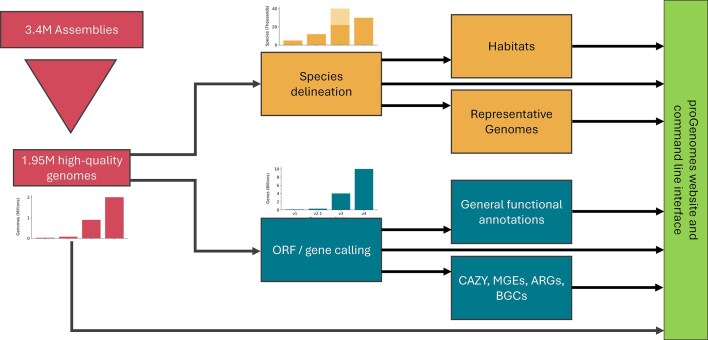

## Introduction

The first microbial genomes were sequenced >30 years ago [1], yet the availability of large-scale genomics data remains a driver for discovery and innovation [2]. Nowadays, the availability of high-quality, low-cost genome sequencing using both short and long reads ensures that high-quality genomes can be assembled for any cultured organism [3]. Nevertheless, to gain biological insight from these ever-growing data sets, scientists need access to high-quality genomes with consistent annotations [4].

Microbial genomes are available from a variety of databases. The NCBI RefSeq database [5] is a prominent example providing access to a wide range of genome sequences, yet it only provides gene names, gene symbols, and EC numbers as annotations for coding sequences. Similar information, often enriched by specific annotations, is available from the PATRIC (Pathosystems Resource Integration Center) database [6], Ensembl Bacteria [7], and the Joint Genome Institute Integrated Microbial Genomes & Microbiomes database [8]. More recently, the AllTheBacteria database has combined *de novo* assemblies of genomes from public repositories with functional annotations [9]. In addition, databases such as SPIRE, MGnify, and motus-db provide millions of metagenome-assembled genomes (MAGs) [10, 11]. The Genome Taxonomy Database (GTDB) [12] is a dedicated, genomics- and phylogeny-based consistent taxonomy covering bacteria and archaea, comprising both reference genomes and MAGs. GTDB resolves many inconsistencies in previous taxonomies and provides an automated way to find and correct submitter errors.

In addition to such taxonomic annotation consistency, functional annotations have the same requirement, as has habitat information. Habitat information in particular is often inconsistent or missing as it depends on submitters. To address this issue, multiple resources linking microbes to environments have been established, including Microbe Atlas Project (MAP) [13] and Omnicrobe [14]. proGenomes4 (prokaryotic Genomes v4) integrates and links to MAP, which uses a comprehensive, annotated 16S ribosomal RNA (rRNA) catalog to link taxa to habitats, which are organized in an ontology.

Another important yet often neglected issue in microbial genomic databases is genome quality. Tools such as CheckM [15, 16] and GUNC [17] provide the means to consistently assess the quality of genomes at scale.

Here, we present the proGenomes4 database, which provides nearly 2 million high-quality bacterial and archaeal reference genomes of isolates (twice as many as in the previous version). proGenomes enables researchers’ direct access to deeply and consistently annotated, high-quality genomes, providing information relevant for many different disciplines including but not limited to microbial evolution, ecology, and clinical and applied microbiology [18–20]. Genome quality is assured by using checkM2 and GUNC [16, 17]. Multiple annotation layers provide both general functional annotations via eggNOG [21] as well as specialized information about e.g. mobile genetic elements (MGEs) [22] and biosynthetic gene clusters (BGCs) [23] for over 8 billion genes. Further, the genomes are linked to other databases and resources, providing comprehensive access to information. proGenomes4 is designed to provide direct and easy access to the data and information needed for comparative analyses of prokaryotic genomes at any scale. The database can be accessed via a newly developed command line interface for bulk downloads and is available at https://progenomes.embl.de/.

### Database construction and characteristics

proGenomes4 is accessible via its website (https://progenomes.embl.de/), which gives users access to all data and enables them to browse the available microbial genomes. By specifying NCBI assembly ID or the taxonomic name of the organism, species or clade in the search bar, users can interactively find and explore information about their desired organisms. In addition, the downloads section allows users to download precomputed bulk files providing e.g. the genomes and annotations of all species representatives. Major upgrades of the underlying computational pipeline and dataset are planned every two years.

### Genome collection

**Figure 1.**
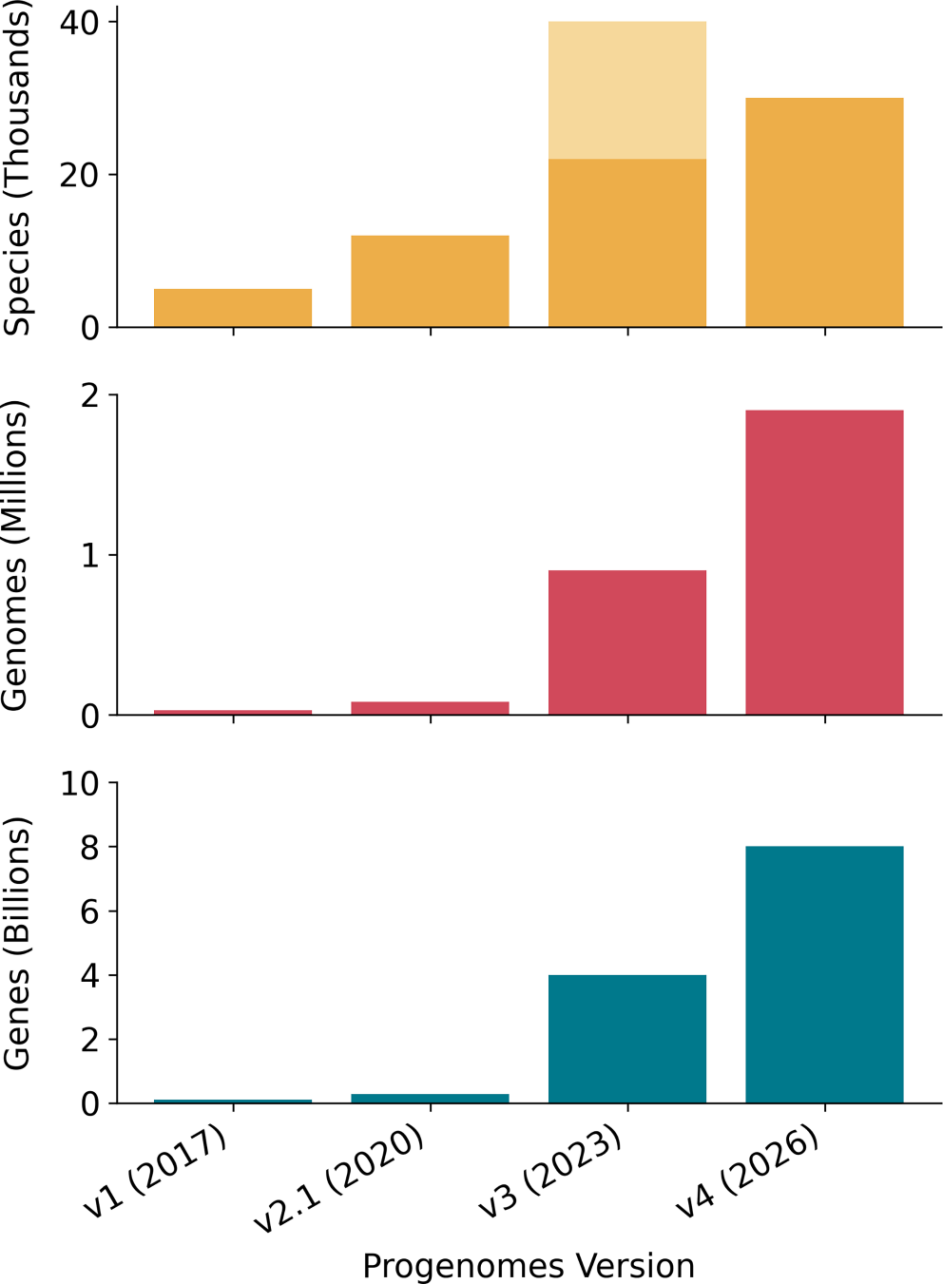
Growth of proGenomes across versions. Overall number of species, high-quality genomes, and genes in proGenomes (2017), proGenomes2 (2020), proGenomes3 (2023), and proGenomes4. The number of species in proGenomes3 is shown with and without MAGs, for proGenomes4 all possible MAGs were excluded.

All 3.1 million bacterial and archaeal genomes available in the NCBI Nucleotide database were downloaded on 3 April 2025 using NCBI Datasets CLI (version 18.4.0) [24]. First, we removed duplicate genomes (i.e. genomes present in multiple NCBI databases under different but linked accessions) and those marked as “suppressed” or “derived from metagenomes.” Open reading frames were predicted for these genomes using prodigal (v2.6.3) (default parameters) [25]. Genomes with a circular, closed assembly were treated as high quality by default. Other incomplete genomes were filtered to only retain high-quality genomes using CheckM2 (v1.1.0) [16] and GUNC (v1.0.6) [17] (CheckM: completeness >90% and contamination <5%; GUNC: contamination <5% and clade separation score <0.45). This quality control step removed 1.2M genomes, with 1.9M high-quality genomes remaining (Fig. 1).

### Delineating species

We used an ANI (average nucleotide identity)-based approach to consistently delineate species [26]. In short, we first preclustered genomes using Mash v2.3 (parameter -d 0.1) [27] under a *single linkage* algorithm, ensuring that all pairs of genomes sharing ≥90% mash similarity are part of the same precluster. Preclusters were then resolved using *average linkage* clustering at ≥95% ANI [calculated using fastANI (1.32) [26]] to obtain species-level clusters.

### Selection of representative genomes

With the steady increase in genomes, there are many species now for which genomes from multiple reference strains are now available, leading to a certain degree of redundancy. While proGenomes4 continues to provide annotations for all high-quality genomes, many applications require nonredundant genome set (e.g. metagenomic read mapping or metagenomics-based strain tracking [28, 29])

As for previous versions, proGenomes4 provides a nonredundant set of representative genomes. Precomputed FASTA files of all representative genomes can be downloaded directly on the proGenomes website.

For the majority of species, we chose representatives by genome quality and citation statistics. For 820 species, however, we utilized a manually curated list of strains that are *de facto* representatives of their respective species, e.g. *Mycobacterium tuberculosis* H37Rv. Otherwise, we filtered each species genome set to only contain complete genomes and chose the most highly cited strain out of those [30]. If a species did not have a single complete genome, the genome assembly with the highest N50 statistic was selected.

### Pan-genomes

Many species in proGenomes4 are represented by many different genomes. To provide users with a simple way to study all genes encoded by a species, we precompiled pan-genomes for every species in the form of a nonredundant gene set. The nonredundant gene sets were generated using mmseqs2 [31] (version: 18) using following parameters: --min-seq-id 0.95 -c 0.90 --cov-mode 0

### Functional annotation

Functional annotations are one main aspect of proGenomes, with the goal of providing accurate, consistent, and broad annotations. General functional annotations were generated using eggNOG-mapper [32] (version:2.1.12). This results in assignments to functionally annotated orthologous groups from eggNOG 5.0 [33]. This broad annotation effort resulted in ~6 billion predicted proteins to be annotated to existing orthologous groups.

Carbohydrate utilization is one of the major sources for microbial energy generation. Hence, there are dedicated tools and databases providing carbohydrate-active enzyme annotations. For proGenomes4, we are utilizing Cayman, which has been shown to be highly accurate [34].

Antimicrobial resistance genes were annotated using Abricate 1.0.1 with three different databases (i.e. vfdb, megares, and deeparg 1.0.4)

We used proMGE to identify MGEs across representative genomes, using recombinases as annotation anchors and pangenome information for determining MGE boundaries [22].

Similar to MGEs, enzymes often function in conjunction with the enzymes encoded by neighboring genes. Such BGCs often encode for the cellular machinery producing ecologically or clinically relevant metabolites. We predicted the presence of BGCs using GECCO (v0.9.8) [23].

### Habitat information

**Figure 2.**
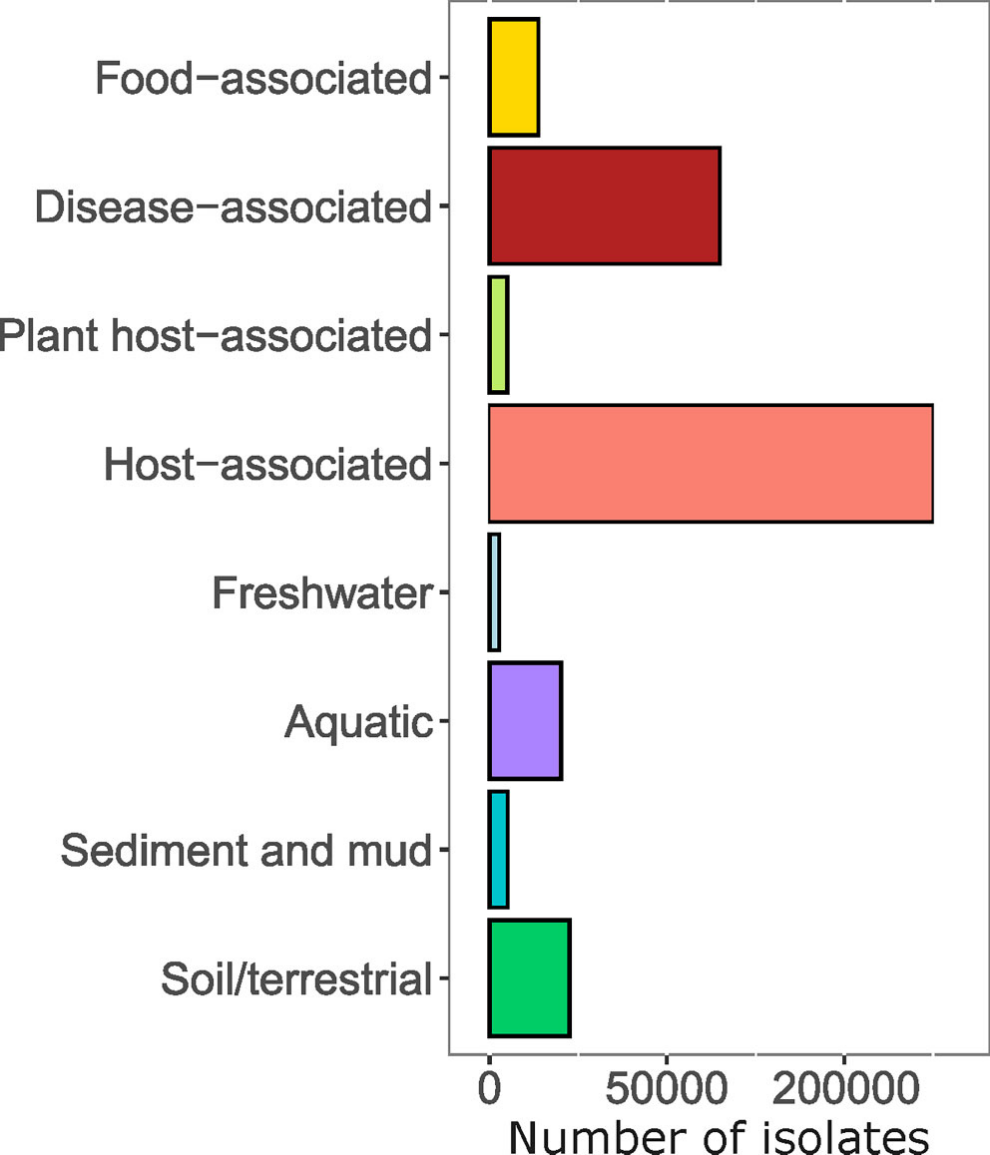
Curated habitat annotations. The number of isolates annotated to the different habitats is shown.

There has been a growing interest in consistent habitat annotations for microbial genomes, both for large scale analyses and classical microbiology applications. proGenomes4 uses both source information (via BV-BRC v3.53.3, accessed on 11 September 2025 [35]) and species detection in environmental sequencing data (via MAP [13]) to provide such annotations. BV-BRC habitat annotation enabled us to annotate 379 068 out of 1.9 M genomes (Fig. 2), which represents a two-fold increase of annotated genomes compared to the previous version of the database (proGenomes3).

To annotate habitats using the MAP, we extracted 16S rRNA genes from genomes and matched them to the sequences in the MAP v3 database using MAPseq [36]. Next, we linked proGenomes species clusters and 98% MAP Operational Taxonomic Units (OTUs) using the mapped 16S sequences. By applying a majority rule, the best matching MAP OTU was identified for each proGenomes species cluster if at least 80% of the 16S sequences of a given proGenomes species cluster were mapped to the same 98% MAP OTU.

### Links to outside databases

Even though proGenomes4 provides many different annotation tracks, dedicated databases often provide details that cannot be mirrored. Instead, we chose to add additional links to outside databases such as NCBI Genome [24], BacDive [37], GTDB [12], and MAP [36] enabling direct access.

### Database design

At its core, proGenomes4 is a PostgreSQL-powered relational database system, which stores all obtained information on the included genomes and their features. The website can directly interact with the database and make the information available to the users. To efficiently access the sequence information (genomes, gene, and protein sequences), indexed FASTA flatfiles are utilized.

### Programmatic access

In this version, we make available a command line tool and Python package to facilitate high-throughput use of the database. Users can easily download genome sets by habitat and genomic datasets. The tool maps the various artifacts in the database to facilitate download by users. It contains two main subcommands: view and download. The first allows users to preview datasets from proGenomes4 in the command line. The download command allows the selection of either datasets or genomic sets as the download target. For genomic sets, it also enables users to select which components to download for each habitat: contigs, genes, and proteins. The functional annotations for all representative genomes are also available via the command line tool.

The command line tool is available at https://github.com/BigDataBiology/progenomes-cli and can be readily installed via pypi.

### Website

proGenomes4 can be accessed via its dedicated website (https://progenomes.embl.de). The genomes of taxonomic groups as well as specI clusters can be accessed easily via a search function. For each genome, we provide the information stored within proGenomes3 as well as direct links to external database entries.

### Future outlook

proGenomes will continue to be developed actively, ocaften in response to user feedback. We aim to add additional annotation tracks in future releases as well as to improve the options for programmatic access introduced in the current release. Further, we are striving to improve integration with other resources and will determine best practices for this purpose.

## Discussion

proGenomes4 offers easy and direct access to over 2 million high-quality genomes, including multiple functional annotation tracks, as well as taxonomic and habitat assignments via a dedicated website. The website further provides links to relevant entries in related databases and direct download of bulk data. proGenomes serves a broad user base ranging from microbiologists interested in well-annotated genomes for strains used for wet-lab experiments to artificial intelligence researchers interested in microbial genome dynamics. Across its different versions, proGenomes has been used as a foundation for multiple widely used resources such as eggNOG, mOTUs, and Spire.

proGenomes4 will continue to be a valuable resource, which we expect to be widely used by a broad range of researchers.

## Data Availability

Progenomes4 can be accessed via its dedicated website (https://progenomes.embl.de). The source code for the command line tool is available at https://github.com/BigDataBiology/progenomes-cli.
